# Macro and micro plastics sorb and desorb metals and act as a point source of trace metals to coastal ecosystems

**DOI:** 10.1371/journal.pone.0191759

**Published:** 2018-02-14

**Authors:** B. Munier, L. I. Bendell

**Affiliations:** Ecotoxicology Research Group, Department of Biological Sciences, Simon Fraser University, Burnaby, B.C. Canada; VIT University, INDIA

## Abstract

Nine urban intertidal regions in Burrard Inlet, Vancouver, British Columbia, Canada, were sampled for plastic debris. Debris included macro and micro plastics and originated from a wide diversity of uses ranging from personal hygiene to solar cells. Debris was characterized for its polymer through standard physiochemical characteristics, then subject to a weak acid extraction to remove the metals, zinc, copper, cadmium and lead from the polymer. Recently manufactured low density polyethylene (LDPE), nylon, polyethylene terephthalate (PET), polypropylene (PP), polystyrene (PS) and polyvinyl chloride (PVC) were subject to the same extraction. Data was statistically analyzed by appropriate parametric and non-parametric tests when needed with significance set at P < 0.05. Polymers identified in field samples in order of abundance were; PVC (39), LDPE (28), PS (18), polyethylene (PE, 9), PP (8), nylon (8), high density polyethylene (HDPE, 7), polycarbonate (PC, 6), PET (6), polyurethane (PUR, 3) and polyoxymethylene (POM, 2). PVC and LDPE accounted for 46% of all samples. Field samples of PVC, HDPE and LDPE had significantly greater amounts of acid extracted copper and HDPE, LDPE and PUR significantly greater amounts of acid extracted zinc. PVC and LDPE had significantly greater amounts of acid extracted cadmium and PVC tended to have greater levels of acid extracted lead, significantly so for HDPE. Five of the collected items demonstrated extreme levels of acid extracted metal; greatest concentrations were 188, 6667, 698,000 and 930 μgg^-1^ of copper, zinc, lead and cadmium respectively recovered from an unidentified object comprised of PVC. Comparison of recently manufactured versus field samples indicated that recently manufactured samples had significantly greater amounts of acid extracted cadmium and zinc and field samples significantly greater amounts of acid extracted copper and lead which was primarily attributed to metal extracted from field samples of PVC. Plastic debris will affect metals within coastal ecosystems by; 1) providing a sorption site (copper and lead), notably for PVC 2) desorption from the plastic i.e., the “inherent” load (cadmium and zinc) and 3) serving as a point source of acute trace metal exposure to coastal ecosystems. All three mechanisms will put coastal ecosystems at risk to the toxic effects of these metals.

## Introduction

Rates of plastic production have increased 20 fold since 1964 which has resulted in an estimated 311 million tonnes of plastics within the ocean as of 2014 [1]. Further estimates are that at current rates of plastic production, by 2050, the total mass of plastics will outweigh the biomass of fish[1]. The occurrence of plastics within our environment has become so pervasive that for geologists it has defined the Anthropocene, an epoch of time where humans are the main forcing agents of geological and biological change [2]. When discovered, plastic materials became integrated into all aspects of a modern human lifestyle. However, the very nature of the plastic which provides all of its multiple uses also leads to their permanent nature and hence accumulation within ocean ecosystems. Further, of the plastics now being generated, by some estimates, only 9% is recycled[3]. The result is possibly one of the greatest environmental challenges we as a society have been presented with; what are the impacts of plastics on ocean ecosystems and once identified, can we reverse or mitigate these negative impacts?

Plastic materials are polymers whose chemical structure allows them to be shaped at elevated temperatures and pressures i.e., the long-chain polymers exhibit “plastic flow” when heated. The plastic polymer can be modified with other materials (e.g., plasticizers, fillers and stabilizers), prior to being processed in a molten state [4]. Plastics have been conveniently described based on size with macroplastics being all plastics greater than 5 mm and microplastics, those particles originating from macroplastics less than 5 mm in size. Microplastics also include plastics that are manufactured less than 5 mm in size (e.g. microbeads) [5].

Vethaak and Leslie [6] have outlined three mechanisms by which persistent plastic waste present significant risks to aquatic ecosystems and humans who rely on these ecosystems; 1) Direct toxicity of the plastic particles themselves e.g., oxidative stress, cell damage, inflammation and impairment of energy allocation functions. 2) Chemical toxicity of the plastic debris. These can include heat stabilizers, UV stabilizers, and plasticizers, processing aids, impact modifiers, thermal modifiers fillers, flame retardants, biocides and smoke suppressors. Heat stabilizers and slip agents are of particular concern as they contain the trace metals, cadmium, zinc and lead and can comprise up to 3% of the polymers composition [7] PVCs also contain phthalate plasticizers to improve performance. PVC objects such as piping can be mechanically broken down into increasingly smaller pieces. By doing so, the chemical toxicity of the tubing becomes increasingly of concern as the smaller particles can be ingested by marine organisms. 3) By acting as substratum, plastic particles provide the vector for pathogenic micro-organisms and parasites (e.g., *Escherichia coli*, *Bacillus cereus* and *Stenotrophomonas maltophila*).

A fourth mechanism and one of equal concern to the direct effects of plastics within aquatic ecosystems is the role they play in the sorption of priority pollutants [8,9] thus providing an alternate means of introducing pollutants into freshwater and marine food webs. Recent studies that have addressed the ability of microplastics to sorb trace metals from aquatic and sedimentary environments have indicated that plastic debris can act both as a sorption site for trace metals [10–12], thus allowing for accumulation, or provide an “inherent” load that could also present a source of toxic metal to aquatic ecosystems [13]. Ashton et al.[10] determined the association of metals with plastic production pellets (PPP), sampled from four beaches in SW England and noted that pellets were enriched with cadmium and lead with PPP’s able to accumulate metals to concentrations approaching those of sediment and algal fragments. Holmes et al. [11] assessed the interactions between trace metals and PPP’s, virgin and aged, under estuarine conditions and concluded that plastic pellets effectively sorb trace metals; short term attributed to adsorption of organic matter and long-term which incorporated the aging of the pellet. Rochman et al.[12] compared the long-term sorption of metals among plastic types in seawater and found that in general all types of plastic tended to accumulate similar concentrations of metals and that over a 12 month study period the concentrations of all metals increased over time and did not reach saturation. Wang et al. [13] however, concludes that toxic metals associated with plastic debris are “inherent” rather than accumulated, with this inherent load presenting toxicology threats to the receiving environments.

Hence, our primary objective was to determine the potential role of both macro and micro plastics in providing a source of the trace metals, zinc, copper, cadmium and lead into intertidal foodwebs. To meet our objective we sampled 9 urban intertidal regions within Burrard Inlet, Vancouver, B.C., Canada for plastic debris. Debris was identified for polymer type and subject to a weak acid extraction. Recovered metal was compared among polymers to identify which polymer had the greatest amounts of extracted metal and thus would pose the greatest risk for introducing toxic metals into intertidal food webs. Our hope is to add to the increasing knowledge base on how plastic debris is impacting our marine environment, in this case by providing another vector for the entry of contaminants into marine ecosystems.

## Methods

### Study site and sample collection

**Fig 1:**
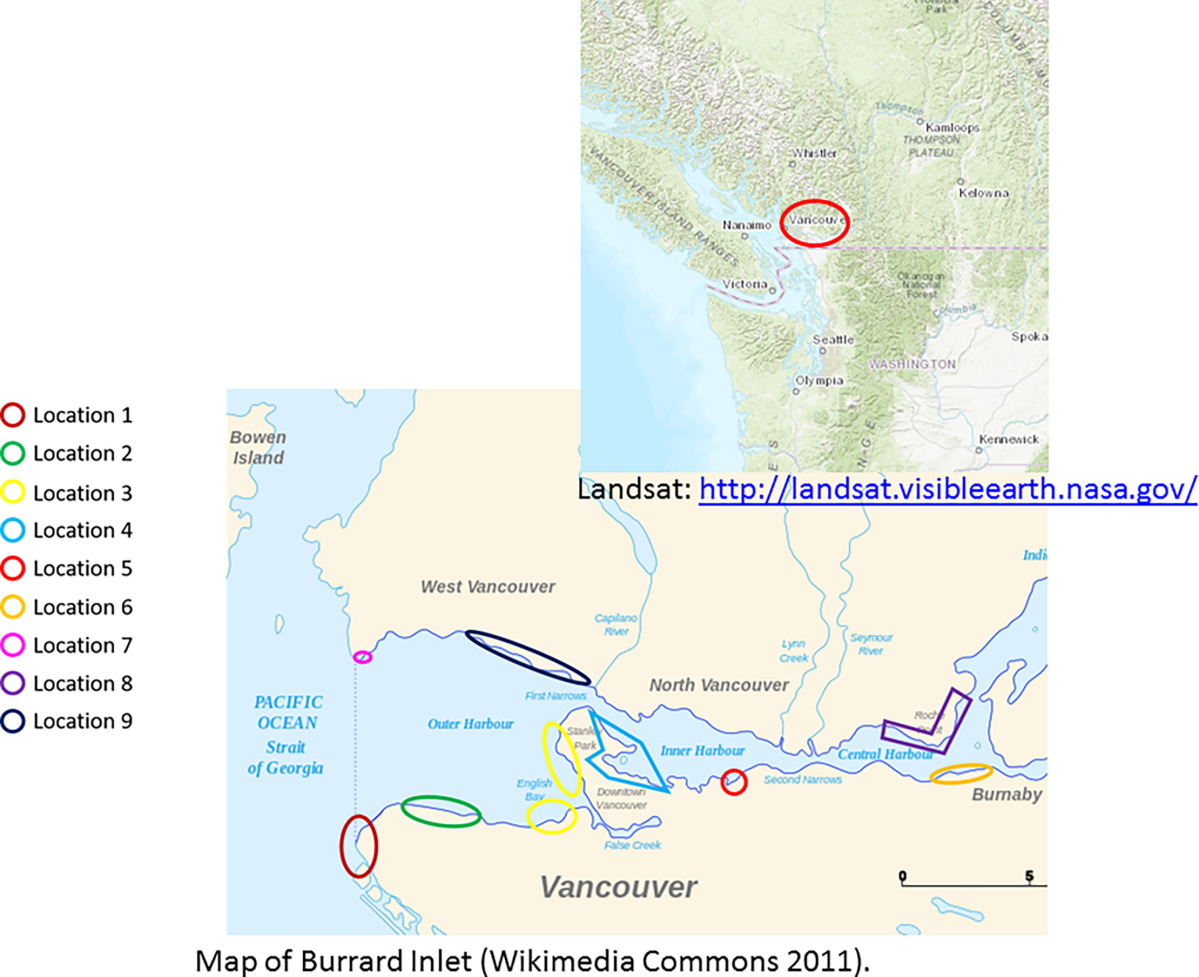
Location of the 9 urban intertidal regions sampled for plastics within Burrard Inlet, Vancouver, B.C. Insert in upper left hand corner indicates location relative to the rest of the Salish Sea, Canada.

Nine beaches within Burrard Inlet (Fig 1) were sampled for plastics. Sampling occurred at low tide such that at least 10 meters of intertidal was exposed. At each site, a 1–5 km line was drawn parallel to the shoreline and a 10 meter line drawn perpendicular to the shoreline and tideline. Within this defined area, every piece of plastic debris that was observed was photographed and placed into a zip lock bag. Twenty six km of beach was surveyed and 150 samples collected. Each item was categorized based on where sampled and object type. No specific permissions were required for the collection of debris from public beaches located in Burrard Inlet, Vancouver BC, Canada. Field studies did not involve endangered or protected species.

### Polymer identification

Each collected plastic was identified for its polymer based on physical tests which included density, flame color and emission characteristics [14–16]

### Trace metal analysis

Field collected samples were weighed and those greater than 1 gram were cut to meet the ca. 1 gram requirement for trace metal extraction. Final sample weights ranged from 0.012 grams to 1.5 grams. Also included in our analyses were six recently manufactured known polymers purchased from a local hardware store. It was assumed that purchased polymers had not been in an environment where exposure to trace metals could have occurred. As we wanted to determine only those metals associated with the surface of the plastics and not those associated with compounds within the plastics, we used a dilute acid extraction. Preliminary extraction experiments where test samples were extracted for one, two and three hours indicated that optimum removal of the metal occurred at 2 hours when gently washed in 10 mL of 10% nitric acid at 30°C. It is important to note that this extraction procedure cannot identify inherit versus sorbed metal associated with the polymer, but rather will the sum of both sources of metal from the plastics. All samples were first rinsed with distilled, deionized water to remove attached materials (e.g., sand) prior to extraction. Once extraction was complete, the 10 mLs was recovered from the digestion flask, tightly covered and stored at 4°C in 15 mL Falcon ™ tubes until analysis. Acid extractions were analyzed for copper, zinc, cadmium and lead via atomic absorption spectroscopy (PinAAcle 500, Perkin Elmer). Standards and blanks were run with each set of analyses to ensure quality assurance and quality control. Blanks were always below limits of detection which were 1 μgL^-1^ for all four metals with precision of the analysis between 3–5%.

### Statistical methods

Statistical analysis was performed using Sigma Plot 12 (SYSTAT Software, Chicago IL). Shapiro-Wilk tests for normality and equal variance tests were applied to ensure that data met the assumptions of the parametric tests. One-way and two ANOVA’s were applied to determine significant differences in trace metal concentrations among polymer types. Where significant differences occurred a Holm-Sidak method was applied to determine where the differences were. If data were not normally distributed, even after transformation, then data was ranked and analyzed by one-way or two-way ANOVA’s on ranks using a Kruskall-Wallace test to determine significance. T-tests on ranked data were applied to determine differences in polymer (all polymer types pooled) metal concentrations between field and recently manufactured samples using a Mann-Whitney Sum test to determine where significant differences occurred. Level of significance was set at 0.05, with 0.1 used to indicate “trends”.

We applied the following statistical analysis;

1. One-way ANOVA for differences in acid extracted metal among recently manufactured polymer samples (nylon, PET, PP, PS and PVC)
2. One-way ANOVA for differences in acid extracted metal among field polymer samples (HDPE, LDPE, nylon, PC, PE, PET, PP, PS, PUR and PVC)
3. Two-way ANOVA for differences in acid extracted metal with field versus recently manufactured and polymer type as the two factors (nylon, PET, PP, PS and PVC).
4. T-tests to determine differences in acid extracted metal between field and recently manufactured polymers.
5. We also where possible tested for differences in color within polymer type. Each field collected polymer was identified by color (i.e. from transparent to black) and differences in acid extracted metal within a polymer type determined by one-way ANOVA. There were only enough samples for PVC and PET for this analysis.
6. Simple linear regressions were applied to determine if amounts of acid extracted metal from field collected samples were dependent on sample weight.

## Results

All data is available in supporting information S1 File.

### Field collection-item identification

**Fig 2:**
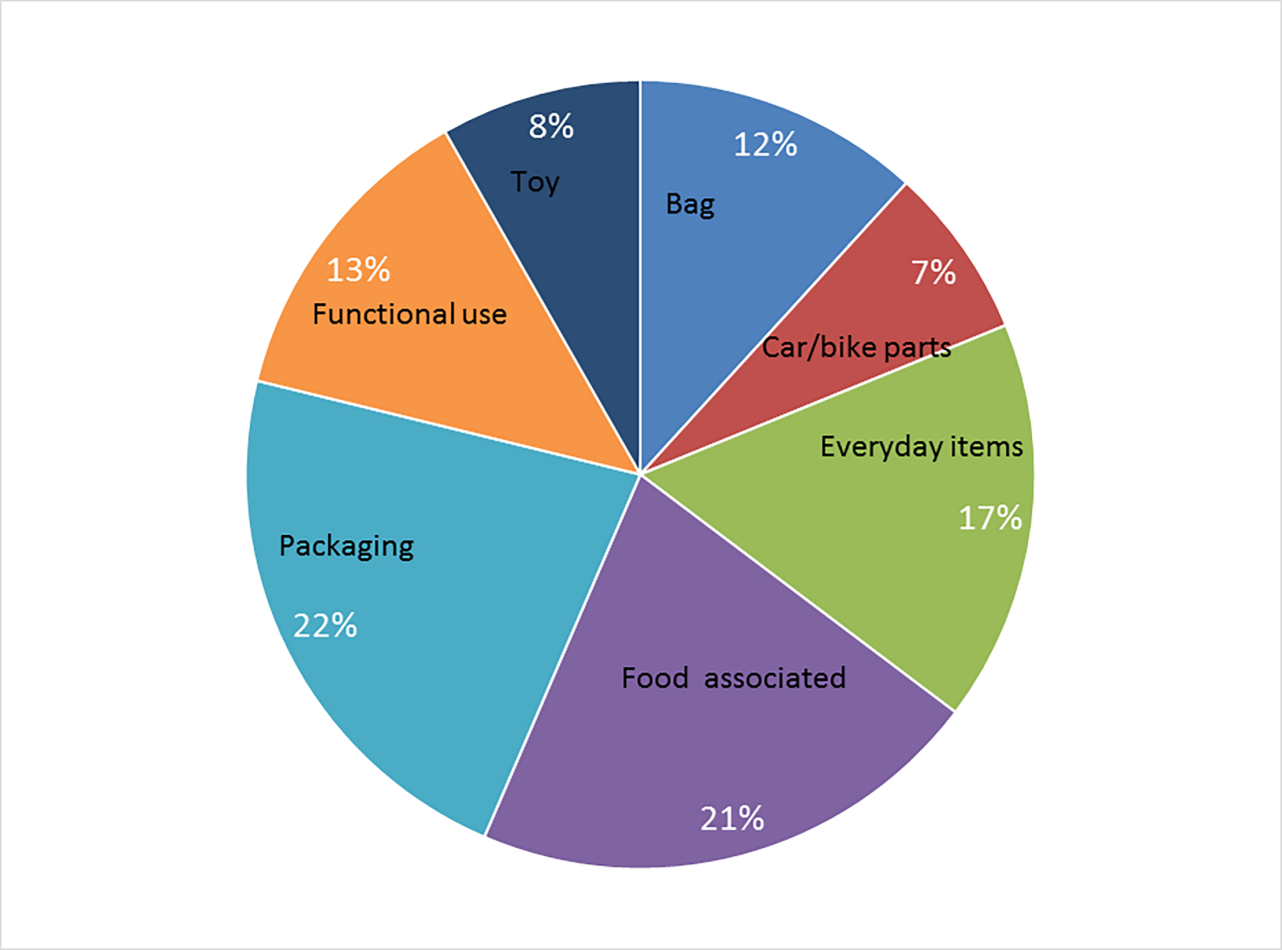
Classification of collected plastics based on original use.

An incredibly diverse number of items were recovered from the urban beaches. One hundred and fifty items were collected of which 144 were plastics. Of the 144, we were able to identify the original use of 85 (Fig 2). These recovered plastics fell into 7 major user groups; bags, car/bike parts, everyday items (e.g., ear buds, glasses), food associated (cup, straw, forks), packaging, functional use (ties, nylon, gloves), and children’s toys (e.g., miniature bicycle). The majority of plastics were wastes associated with food consumption and packaging. Unlike other shore line clean up initiatives [17] that find that the main items collected are cigarette butts, food wrappers and plastic bottle caps, the majority of collected items only occurred once. An important aspect of our collection was that items were both greater and less than 5 mm with some just at the 5 mm limit that distinguished macro from micro plastics. Hence, collected samples represented the transition of macro plastics to micro plastics.

### Polymer Type; field samples

Of the 144 items, 12 polymers were identified. Polymers in order of abundance were; polyvinyl chloride (PVC, 39), low density polyethylene (LDPE, 28), polystyrene (PS, 18), polyethylene (PE, 9), polypropylene (PP, 8), nylon (8), high density polyethylene (HDPE, 7), polycarbonate (PC, 6), polyethylene terephthalate (PET, 6), polyurethane (PUR, 3) and polyoxymethylene (POM, 2). Also identified were rubber (2), amino plastics (1), and nitrile rubber (NBR 1) with 6 unknowns. Not surprisingly, six of the eight most common types of synthetic organic polymers commonly found in households include LDPE, HDPE, PP, PVC, PS and nylon with these polymers accounting for 81% of all samples collected.

### Acid extracted metals from polymers; recently manufactured samples

Polymers purchased from a local hardware store included PVC, nylon, PP, PET, PS and LDPE. Amounts of metal extracted from recently manufactured polymers, PVC, nylon, PP, PET, PS and LDPE are presented in Table 1.

**Table 1:** Concentrations (μgg^-1^ dry weight of polymer) of cadmium, copper, zinc and lead recovered by a weak acid extraction from “recently manufactured” polymers. Values are means of 3 with standard deviations.

		Cadmium	Copper		Zinc		Lead	
Polymer	Mean	SD	Mean	SD	Mean	SD	Mean	SD
PVC	0.42	0.08	3.81	1.48	4.3	1.17	2.67	1.5
Nylon	0.4	0.02	2.93	0.23	10.15	0.79	0.77	1.33
PP	0.37	0.06	4.17	1.8	5.66	0.69	2.16	2.48
PET	0.43	0.06	6.99	2.85	10.48	4.31	2.59	0.71
PS	0.42	0.08	3.71	0.47	8.85	1.6	3.85	1.05
LDPE	1.77	0.62	47.53	34.31	604.24	238.07	52.16	17.68

One-way ANOVAs indicated that for cadmium, copper and lead, amounts of metal recovered from the polymers were not different (P >0.05; LDPE was excluded due to the very high values of acid extracted metal). The exception was for zinc, with nylon and PET both having greater amounts of extracted metal as compared to PVC, but not for PP and PS (F = 4.88; P = 0.019).

### Acid extracted metals from polymers; field samples

**Fig 3:**
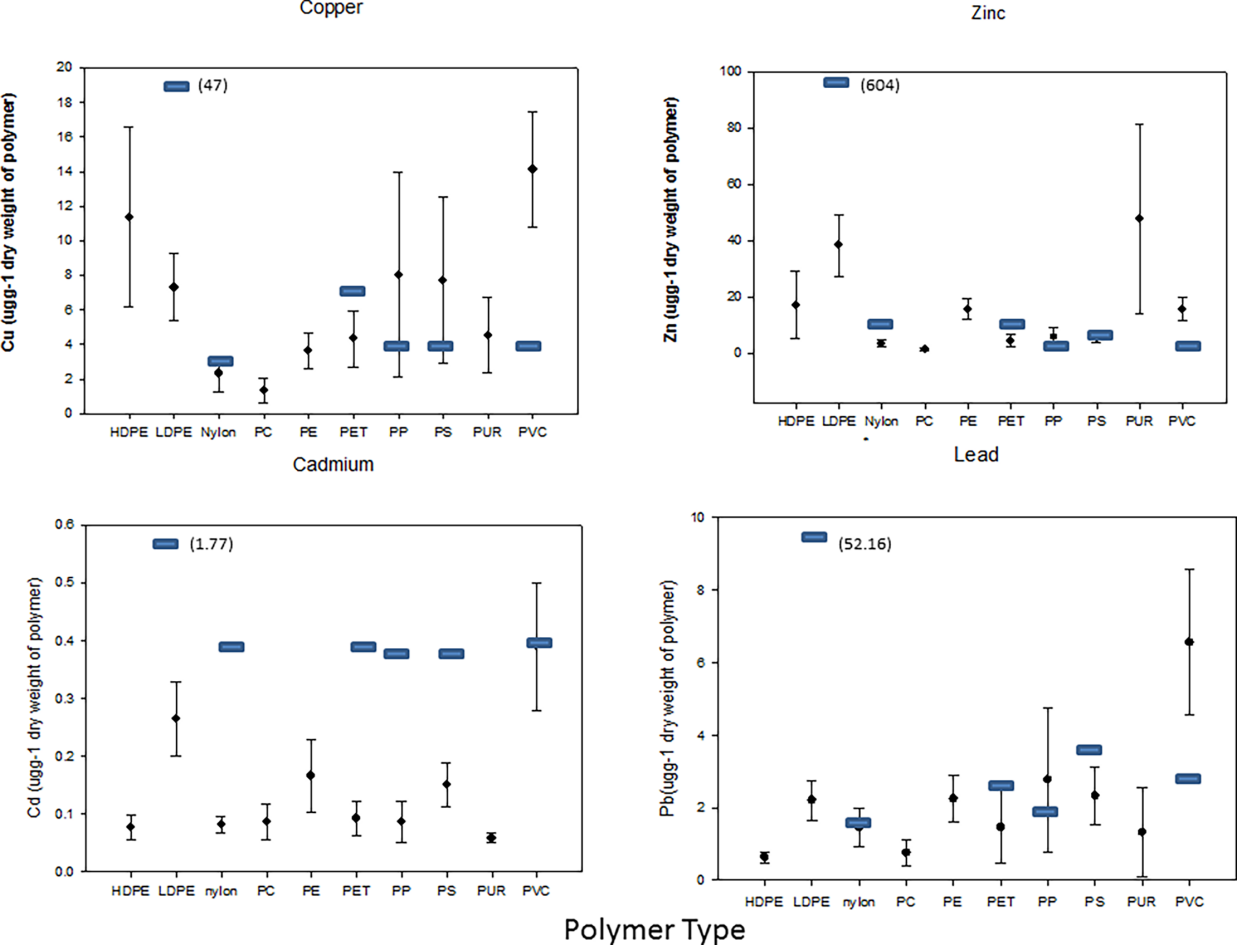
a, b, c and d. Amounts of metal extracted from 10 polymers collected from 9 urban intertidal regions, Burrard Inlet, Vancouver, B.C. Canada; a) copper, b) zinc, c) cadmium and d) lead. Values are in μgg^-1^ dry weight of polymer and are means with 1 standard deviation. Metals extracted from recently manufactured polymers are over-laid with blue bars for comparison. Two additional polymers were identified however; the number of samples was less than 3 so they were not included in the statistical analyses.

One-way ANOVA among polymer types for copper indicated a significant difference (F = 2.448; P = 0.014), with PVC having greater copper concentrations as compared to nylon and PC (F = 1.3; P< 0.05) (Fig 3A). Zinc also differed among polymers (F = 7.183; P < 0.001) with LDPE having greater concentrations as compared to PC, PS, nylon, PP, PET and PVC. PC had the lowest amounts of acid extracted zinc as compared to PE, PUR and PVC (Holm-Sidak, P < 0.05, Fig 3B). One-way ANOVA’s for cadmium and lead among polymer type indicated that except for PVC which was greater than PP for cadmium (F = 2.84, P = 0.005, Fig 3C) and greater than HDPE and PC for lead (F = 2.51, P = 0.012, Fig 3D) amounts of acid extracted metal were similar among polymers.

### Differences in acid extracted metal; recently manufactured versus field polymers

When entered into a two-way ANOVA with ID (field versus recently manufactured) and polymer type as the two factors, metal concentrations among polymers were not different, however, amounts of extracted metal was source dependent i.e., either field or recently manufactured (Table 2). (Only those polymers which included both recently manufactured and field samples, nylon, PET, PP, PS and PCV were entered into the two- way ANOVA. Due to the high amounts of sorbed metal recovered from the LDPE, this polymer was excluded from the two-way ANOVA).

**Table 2:** Results of the two-way ANOVA with ID and polymer type as the two factors. F and P are provided for each factor and their interactions. ID is the source of the polymer, either field or recently manufactured.

	Source of Variarion		F	P	Notes	
Copper	ID		4.5	0.037	log(10) transformed	
	polymer		1.1	0.361		
	ID*polymer		0.62	0.648		
Zinc	ID		10.33	0.002	log(10) transformed	
	polymer		0.334	0.845		
	ID*polymer		1.43	0.231		
Lead	ID		9.12	0.004	non-normal	
	polymer		0.52	0.72	data ranked	
	ID*polymer		0.57	0.681		
Cadmium	ID		41.9	0.001	non-normal	
	polymer		0.73	0.57	data ranked.	
	ID*polymer		0.5	0.739		

When all polymer types were pooled for recently manufactured and field samples, a Mann-Whitney Sum test indicated that field polymers contained greater amounts of copper and lead, whereas recently manufactured polymers had greater amounts of zinc and cadmium (Table 3). Differences in field and recently manufactured polymers for copper and lead were driven primarily by amounts of metal extracted from PVC (Fig 3A and 3D).

**Table 3:** Results of the T-test between recently manufactured and field collected polymers. As data was non-normal, a Mann-Whitney Sum test on ranks is presented. Means with SE values are provided although data was ranked for statistical analysis.

Copper		Mean	SE	P
	Field	7.3	1.6	0.1
	Recently	4.3	0.5	F>RM
	Manufactured			
Zinc				
	Field	7.5	1.3	0.009
	Recently	7.9	0.8	RM>F
	Manufactured			
Lead				
	Field	3.4	0.9	0.014
	Recently	3	0.3	F>RM
	Manufactured			
Cadmium				
	Field	0.25	0.06	0.001
	Recently	0.4	0.015	RM>F
	Manufactured			

### Differences in metal desorption, color and weight

Because metals such as cadmium and zinc are used extensively in paint pigments [18], especially for the color red, we determined if color affected amounts of metal recovered from two polymers, PET and PVC. Colors entered into the ANOVA were; transparent, pink red, orange, green, white, yellow, blue, grey and black. One-way ANOVA’s with color as the dependent factor indicated that amounts of extracted metal were not color dependent (F = 0.6; P> 0.05). Simple regression also indicated that the size of the sample did not influence amounts of metal recovered for zinc, copper or lead (R^2^<0.2; P> 0.05). However, cadmium did show a slight relationship (R^2^=0.6; P < 0.05) with the two smallest samples desorbing the greatest amounts of metal and likely related to the surface area to volume ratio of the sample. This could be of importance for cadmium in that as compared to lead, copper and zinc, cadmium is an important additive during polymer formation. Of the four metals it could be more liable, thereby presenting a greater risk to aquatic environments.

### Collected samples with acutely toxic amounts of acid extracted metal

**Fig 4:**
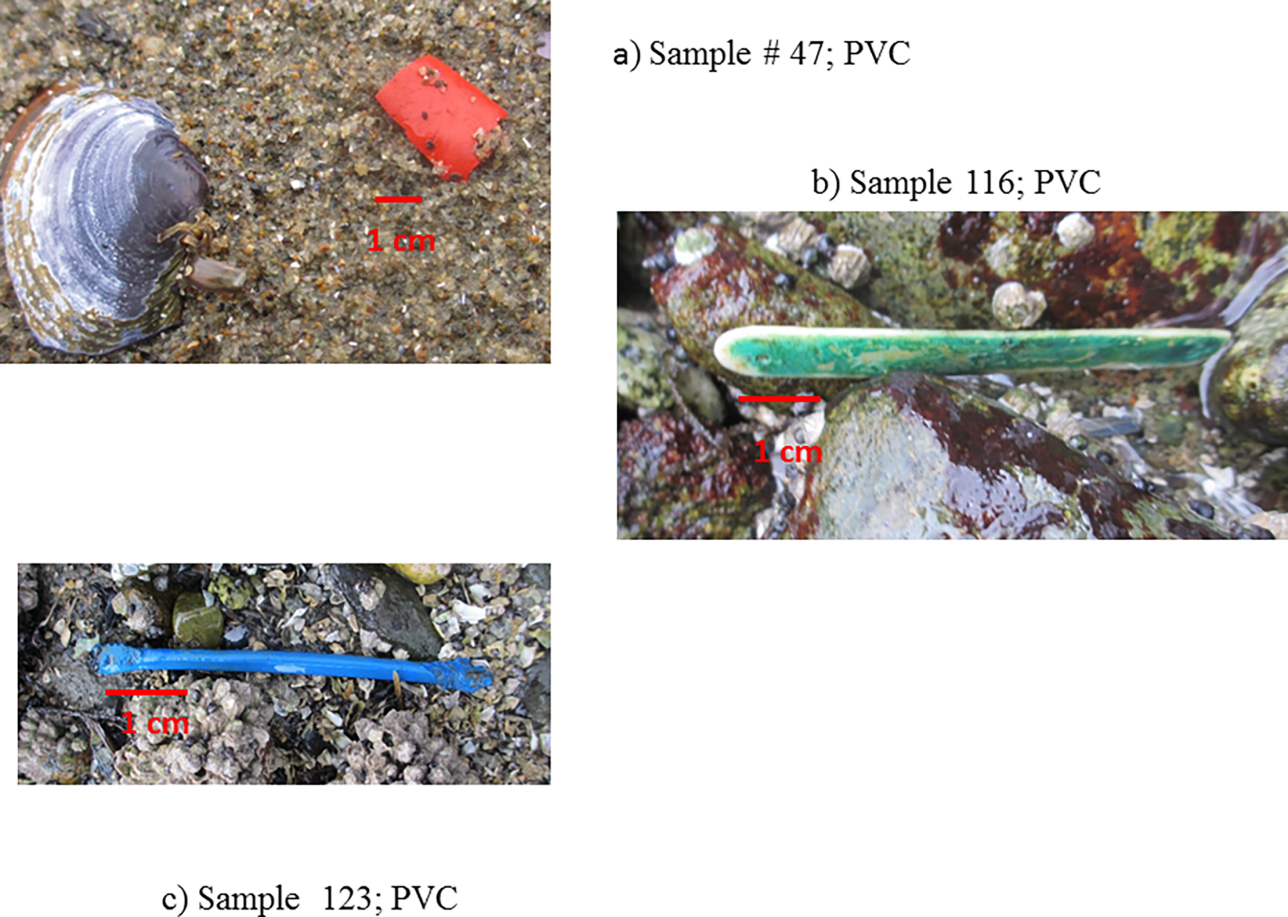
a, b and c. Items collected from intertidal regions of Burrard Inlet, Vancouver B.C., Canada with high concentrations of extracted metal. 4a) unknown, 4b) unknown, 4c) tampon applicator.

Perhaps the finding of most concern was the number of debris items, n = 5, that contained extremely high concentrations of metal Fig 4A, 4B and 4C, Table 4). One sample in particular, #47 (Fig 4A), contained over three orders of magnitude the concentrations of extracted metal as compared to all other samples.

**Table 4:** High metal concentrations recovered from 5 field samples collected from the intertidal regions of Burrard Inlet, Vancouver B.C. Canada. Note units are in mgg^-1^.

Metal	Sample Number	Polymer	mgg^-1^ metal
Copper	116	PVC	12.18
	47	PVC	0.188
	134	LDPE	0.16
Zinc	47	PVC	66.9
	123	PVC	15.57
Lead	47	PVC	698
cadmium	47	PVC	0.09
	61	PS	0.02

The green color of sample 116 (Fig 4B) suggests a copper compound of some sort although its exact origin is unknown. Sample 123 (Fig 4C) was identified as a tampon applicator. Sample # 47 (Fig 4A) is unknown but the high concentrations of metals especially lead could suggest an item related to munitions or explosives.

## Discussion

A random collection of plastics both macro and micro collected from 9 urban intertidal regions revealed an astonishing range in diversity of items reflecting our human culture. Items included children’s toys, bicycle parts, personnel hygiene items and food packaging. Despite the diversity of items, of the 12 polymers identified, ca. 50% of the collected samples were PVC and LDPE.

Using a weak acid extraction our objectives were to determine of the polymers identified, which would pose the greatest risk with respect to the introduction of trace metals into benthic food webs. We assumed that the extraction would remove only those metals loosely associated or surface sorbed to the polymer. Based on a comparison of amount of metal extracted from field collected versus recently manufactured polymers, plastics debris notably PVC, will be sites of sorption for copper and lead, and by contrast an inherent source of zinc and cadmium. PVC was the most abundant polymer recovered from the intertidal amplifying its role in providing a vector for the entry of metals into marine food webs. Also found were 5 samples which contained extremely high concentrations of trace metals.

The greater amounts of extracted cadmium and zinc found for the recently manufactured samples are likely related to the polymer manufacturing process. The International Cadmium Association[19] report that cadmium-bearing stabilizers are used to retard the degradation processes which occur in PVC and related polymers on exposure to heat and sunlight. Cadmium in the form of stearate or laurate is incorporated into the polymer before processing and can account for 0.5–2.5% of the final polymer compounds. Similarly and as noted previously zinc as zinc stearate at amounts up to 3% is commonly used as a plastics stabilizer. This equates to 300 μgg^-1^of zinc and cadmium being introduced into marine ecosystems by polymers such as PVC.

Of note were the order of magnitude greater concentrations of metals extracted from the recently manufactured LDPE as compared to all other polymers. We used recycled new plastic bags as our source of LDPE, without any coloring. Imhof et al. [18] reports for recently manufactured plastic bags of which two were comprised of PET, both non pigmented and pigmented concentrations ranges of 0.15–373, 1.42–80 and ND to 43 μgg^-1^ for copper, zinc and lead respectively. Cadmium was not detected. We found for recently manufactured white plastic bags concentrations of 47, 604, 52 and 1.7 μgg^-1^ for copper, zinc, lead and cadmium respectively. With the exception of cadmium, concentrations of recovered metal are similar from the two sources of polymers, that is, values were equally as great. This poses an interesting finding in that it could be that inherent metals within recycled materials and associated paints are much more liable as compared to non-recycled materials and this finding warrants further study.

The studies of Rochman et al.[12] have found that the long-term sorption of metals is similar among plastic types. Using recently manufactured samples of PET, HDPE, PVC, LDPE and PP, these authors measured the accumulation of metal over a 12 month period at three locations in San Diego Bay, USA. The final average concentrations for all polymers at the end of the 12 months were 4.16, 3.8 and 0.8 μgg^-1^ for zinc, cadmium and lead respectively. Copper was not determined. Values for zinc, cadmium and lead are within the range of what we found in our study. By contrast, Wang et al.[13] have recently concluded that the majority of metals associated with plastics debris are derived from an “inherent load”. Their conclusions were based on data from the long-term sorption of metals by microplastics and a comparison of metal burden among microplastics, macro-litters and fresh plastic products.

We used a weak acid extraction of 10% nitric acid, similar in concentration to extractions that are used to estimate metal bioavailability from sediment components such as iron oxides and organic matter [20]. Amounts of metal extracted from the polymers were similar to or greater than that recovered from the bioavailable fraction sediments [20]. It is feasible then that amounts of metal recovered from the plastics will be bioavailable and hence a source of metal to those organism that ingest plastic debris as food items. Our findings suggest then, that plastic debris can be both source (inherent load) and sink (sorption) for trace metals, providing two chronic routes for the entry of trace metals into aquatic food webs; via water for zinc and cadmium and through ingestion for copper and lead. Of great concern was the discovery of plastic items, some less than 5 mm that contained very high concentrations of metals. These items contained copper, lead, zinc and cadmium at levels that would be considered point sources of contaminants into intertidal ecosystems.

In sum, depending on the metal and the type of polymer, plastics will have three modes of action affecting trace metals in intertidal ecosystems, 1) direct release into the overlying water column as a consequence of leaching from the plastic itself, i.e. for cadmium and zinc, 2) entry into benthic food webs through ingestion of plastic particles, notably for PVC, that have accumulated metal i.e., copper and lead and 3) as a point source of toxic metal. All three mechanisms will present toxicological threats to our coastal ecosystems.

## Supporting information

S1 File Metal concentrations for all plastics collected from 9 intertidal regions in Burrard Inlet, BC, Canada. (XLSX)
